# Physiotherapy Rehabilitation Towards Foot Drop in a Paediatric Case of Iatrogenic Sciatic Nerve Palsy: A Case Report

**DOI:** 10.7759/cureus.51771

**Published:** 2024-01-06

**Authors:** Akshaya Saklecha, Pallavi Harjpal, Ragini Dadgal

**Affiliations:** 1 Department of Neuro Physiotherapy, Ravi Nair Physiotherapy College, Datta Meghe Institute of Higher Education and Research, Wardha, IND

**Keywords:** case report, physiotherapy rehabilitation, foot drop, sciatic nerve injury, paediatric injection palsy

## Abstract

In this report, we are presenting a case of injection nerve palsy in a three-year-old child whose parents visited with the complaint of weakness of the left lower limb, inability to walk, and difficulty in performing lower limb movements after taking diphtheria-tetanus-pertussis (DPT) vaccination in the gluteal region by intramuscular route. The child exhibited a foot drop on his left leg and a high step gait when examined. Nerve conduction velocity was performed, which revealed pure motor axonal mononeuropathy involving the left sciatic nerve. She was diagnosed with a left sciatic nerve injury from a foot drop and was referred to physiotherapy. With the proper exercise protocol, physiotherapy rehabilitation began. We report that after rehabilitation, she showed improvement in the strength of the lower limb and gait pattern. As a result, physiotherapy is critical in improving a patient's gait pattern, ensuring early and rapid recovery, and treating the condition's clinical manifestations. This case study concludes that physiotherapy rehabilitation for injection palsy in a three-year-old female child with foot drop led to improved lower-limb strength, which assisted the patient in ambulation and prevented other deformities.

## Introduction

Nerve injury is a typical consequence of intramuscular injection, the most commonly impacted nerve being the sciatic nerve, particularly in paediatrics [1]. According to the WHO, 50 percent of the 12 billion injections given worldwide each year are given in a hazardous manner, and 75 percent are given inappropriately [2]. Sciatic nerve injury occurs due to various causes, including badly placed intramuscular injections in the gluteal region, compression within the pelvis by a neoplasm or foetal head, dislocation of the hip joint, fractures of the pelvis and femur, etc. Almost 90% of patients experience an early onset of symptoms; 10% experience a late onset after injection, which occurs in minutes to hours [3]. It is sensory-motor palsy. The sciatic nerve is usually damaged after an intramuscular injection and the gluteal region is a common site of injection. The presenting features are loss of movement, loss of sensations, being unable to stand, gait abnormalities, and foot drop deformity. Nerve conduction velocity (NCV), an electrodiagnostic study, is beneficial in the detection of nerve damage [4]. The sciatic nerve is made up of two anatomically distinct components, the common peroneal and tibial nerves. The common peroneal component is more frequently affected than the tibial. A complete lesion of the sciatic nerve is rare. The extensors and evertors of the foot are supplied by the common peroneal nerve. A paralysis of these muscles results in a foot drop [5].

Foot drop, often called drop foot, is a term used to describe the inability to lift the front portion of the foot. It leads to gait impairment, in which the patient walks dragging their forefoot on the ground and exhibits a high stepping gait. The most prevalent cause is the compression of a peroneal nerve, a branch of the sciatic nerve [6]. Based on the level of muscle weakness and muscle paralysis, a foot drop can sometimes be temporary or permanent, and this can involve either one or both feet [7]. Rather than being an illness within itself, it is typically indicative of a bigger condition. The patient walks with a high-step gait, i.e., while walking, they have to lift the foot high in order to clear the ground [8]. The muscles that will be paralysed are the biceps femoris and the semi-membranous, semi-tendinous, and hamstring parts of the adductor magnus. Tibialis anterior, extensor hallucis longus, extensor digitorum longus, and peroneus tertitus are dorsiflexors, while peroneus longus and peroneus brevis are evertors. Thus, below the knee, all the muscles will be paralysed. The condition is managed through medical, surgical, and physiotherapy management [9]. Physiotherapy helps to improve the patient's muscle strength, prevent deformity, and improve quality of life [10]. Subsequently, physiotherapy plays a crucial role in the early recovery of the patient and the management of clinical presentations. We describe a case of injection palsy in a three-year-old girl whose parents presented her with a complaint of weakness of the left lower limb, inability to walk, and difficulty in performing lower limb movements after taking diphtheria-pertussis-tetanus (DPT) vaccination in the gluteal region by the intramuscular route.

## Case presentation

Patient information

A three-year-old female child complained of weakness, inability to use the left lower limb, inability to walk, and difficulty conducting dorsiflexion of the left foot. She appeared to be all right two months ago. On October 30, the diphtheria-pertussis-tetanus (DPT) vaccination dose was scheduled and administered to the left buttock region through the intramuscular route. After the next day of vaccination, she felt a sudden weakness in her left lower limb, was unable to bear weight on that leg, and then slowly, also had difficulty using the left lower limb. With these complaints, her parents brought her to the tertiary healthcare centre in Sevagram, Maharashtra, India, where an NCV investigation was carried out that revealed injection palsy of the sciatic nerve after vaccination. She took the prescription for it, and the symptoms subsided. A few days later, she had greater trouble standing and walking, along with weakness in the left lower limb. For this reason, her parents decided to take her to Acharya Vinoba Bhave Rural Hospital (AVBRH), Wardha, Maharashtra, India, in December 2021. Investigations were conducted and she was diagnosed with a left sciatic nerve injury (injection palsy) from a foot drop and referred for physiotherapy. On December 13, 2021, with the proper protocol, physiotherapy and rehabilitation began. Table 1 depicts a summary of the timeline of events that happened.

**Table 1:** Timeline DPT: Diphtheria-pertussis-tetanus; AVBRH: Acharya Vinoba Bhave Rural Hospital

Timeline	Events
30 October, 2021	She was administered DPT vaccination.
1 November, 2021	The patient felt weakness in the left lower limb and had difficulty walking.
18 November, 2021	The patient visited a tertiary healthcare centre in Sevagram, Maharashtra, India.
10 December, 2021	The patient visited AVBRH, Wardha, Maharashtra, India, and investigations were performed and a diagnosis was made.
13 December, 2021	Physiotherapy rehabilitation was begun.
12 January, 2022	Physiotherapy was completed and follow-up was done.

Clinical findings

**Figure 1:**
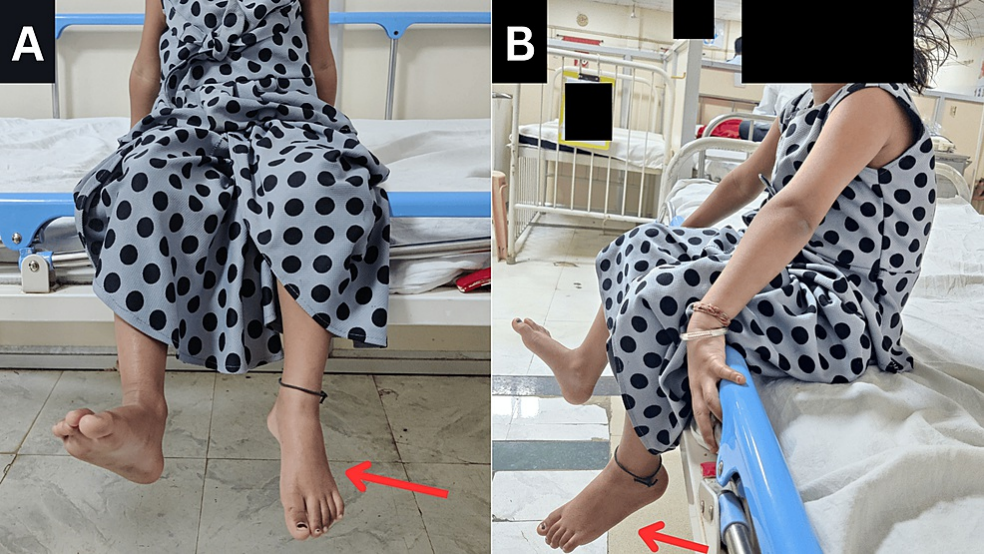
While the patient was trying to dorsiflex both ankles, she was unable to dorsiflex the left ankle, suggesting a foot drop on that side The arrow shows a foot drop on the left side A: Anterior view; B: Lateral view

Before examination, verbal consent was obtained from the patient's parents. In the supine posture, the patient was assessed at both the anterior superior iliac spines (ASISs) at the same level. Along with a blood pressure of 110/70 mmHg, a respiration rate of 30 breaths per minute, and height and weight of 91 cm and 11 kg, respectively were noted. The patient's vital signs were normal. Her higher mental functions and cranial nerves were found to be normal during a neurological examination. The hip was externally rotated with the knee in extension and the ankle in eversion and plantarflexion, and a drop foot was seen on the left side during observation depicted in Figure 1.

On the Medical Research Council scale, the power of her left hip muscles was grade 3/5, her knee flexors had been reduced to a grade 3/5, and her ankle dorsiflexors were grade 0/5 and ankle plantar flexors were grade 0/5 as shown in Table 2. 

**Table 2:** Power in the lower limb according to MRC grading MRC: Medical Research Council Muscle power grading according to MRC: 0- No contraction, 3- Full range of motion against gravity, 5- Full range of motion against maximal resistance

Joint	Power	Right (unaffected limb)	Left (affected limb)
Hip	Flexors	5	3
	Extensors	5	3
	Abductors	5	3
	Adductors	5	3
	Internal rotators	5	3
	External rotators	5	3
Knee	Flexors	5	3
	Extensors	5	3
Ankle	Dorsiflexors	5	0
	Plantarflexors	5	0

There was a normal sensation in both the lower legs. The deep tendon reflexes of the left lower limb were diminished as depicted in Table 3. She had a high stepping gait as evidenced by an abnormal lift of the left lower limb while walking and lifting the knee high, affected foot slaps on the ground. There was a weakness of ankle dorsiflexion, which was compensated by exaggerated hip and knee flexion. The nerve conduction test conducted and showed that pure motor axonal mononeuropathy prominently involved the left sciatic nerve.

**Table 3:** Deep tendon reflexes ++: Normal reflex; +: Diminished reflex

Reflexes	Right	Left
Knee jerk (L3-4)	++	+
Ankle jerk (S1)	++	+

Diagnostic assessment

A neurological examination was carried out, which showed the signs of chances of injury to the sciatic nerve. Electrodiagnostic testing was conducted, which involved NCV and strength-duration curve. NCV revealed the pure motor axonal mononeuropathy prominently involving the left sciatic nerve. The strength-duration curve was plotted for the tibialis anterior muscle, which showed the status of denervation. As the patient was paediatric, there was a limited investigation performed. And she was diagnosed with a left sciatic nerve injury (injection palsy) having a foot drop of the left leg. A diagnosis was confirmed based on the findings of NCV. The NCV electrodiagnostic testing was consistent with the findings of pure motor axonal mononeuropathy prominently involving the left sciatic nerve and subsequent diagnosis of injection nerve palsy with foot drop were noted.

Therapeutic interventions

The therapeutic goals were to improve the strength of lower limb muscles, to prevent further deformity, to promote reinnervation of nerve, to increase flexibility and mobility, and to improve the gait biomechanics. From week zero to week two, the first priority was to provide foot orthosis to the left lower limb to facilitate correct gait mechanics, which would maintain the foot in the neutral position of dorsi and plantar flexion. Soft tissue stretching was avoided. Before giving the splint, we provided the proper education to the parents regarding the positioning, proper usage, and maintenance of the splint. It improves gait mechanics, decreases falls, and helps to minimize other secondary complaints. Tendon Achilles stretching was given with a 30-second hold with three sets twice a day. Stretching exercises are an excellent treatment for foot drop. Passive range of motion to all the lower limb joints was given twice a day of 10 repetitions. Electrical stimulation to the dorsiflexor muscles was given by using the faradic type of current for a duration of five minutes. Strength training was initiated for lower limb muscles, i.e., knee flexors and extensors with the help of a 250-gram weight cuff for 10 repetitions. At the end of two weeks, the patient was able to dorsiflex the ankle in gravity eliminated plane, i.e., grade 2 according to the Medical Research Council Scale of Power.

In weeks two to four, all the previous exercises were continued in these phases along with strengthening exercises and gait training. Assisted active movements were started for 10 repetitions twice a day. Strengthening exercises were continued, with the intensity and duration gradually increasing. Marble picking up exercises were started in which the patient was asked to pick up the marbles using the affected foot toes from the container and again place them in the container while sitting in a chair 10 times a day. Toe curls exercise five times a day was initiated and then progressed by putting the weight at the end of the towel, which provides resistance. Toe-to-heel rock exercises were initiated, in which the patient was asked to stand in front of the table and hold it for support. Then the patient was asked to rock the weight forward and bring her weight forward and up onto her toes. She was asked to continue to hold for five seconds and then, shift her weight back onto her heels and lift her toes off the ground. She was asked to continue to hold for five seconds. Ten repetitions of these were performed. The ball lift exercise was done 10 times with a hold of 5 seconds. The patient was asked to sit in the chair with both feet flat on the floor and to hold the object between her feet while slowly lifting it with her legs extended. Gait training was initiated. Initially, it was done with parallel bars with footmarks. The patient was asked to walk with a wide base of support then sideways walking and forward walking. Verbal commands regarding changing directions and rotation were given. Progression was made, i.e., she was asked to walk outside the parallel bars. Further, a home exercise program was given. We provided the patient with home rehabilitation exercises with video-based instruction and all exercise instructions to her parents. Table 4 depicts the week-wise exercise protocol.

**Table 4:** Week-wise exercise protocol

Duration	Physiotherapy intervention
Week 0-2	Foot orthosis to the left lower limb
	Tendo-Achilles stretching
	Passive range of motion to both lower limb
	Electrical stimulation to the dorsiflexor muscles
	Strength training for lower limb muscles
Week 2-4	Assisted active movements
	Strengthening exercises
	Marble picking up exercise
	Toe curls exercise
	Toe-to-heel rock exercises
	Ball lift exercise
	Gait training

Follow-up and outcome of interventions

The follow-up was taken on the fourth week of the rehabilitation. Table 5 shows the findings of strength of lower limb muscles and the Pediatric Balance Scale pre- and post-rehabilitation.

**Table 5:** Pre- and post-rehabilitation findings of power in the left lower limb according to the MRC grading and Pediatric Balance Scale MRC: Medical Research Council Muscle power graded according to the MRC scale: 0- No contraction, 3- Full range of motion against gravity, 4- Full range of motion against minimal resistance, 5- Full range of motion against maximal resistance

Joint	Power	Pre-rehabilitation (Day 1)	Post-rehabilitation (on fourth week)
Hip	Flexors	3/5	4/5
	Extensors	3/5	4/5
	Abductors	3/5	4/5
	Adductors	3/5	4/5
	Internal rotators	3/5	4/5
	External rotators	3/5	4/5
Knee	Flexors	3/5	4/5
	Extensors	3/5	4/5
Ankle	Dorsiflexors	0/5	3/5
	Plantarflexors	0/5	3/5
Outcome measure		Pre-rehabilitation	Post-rehabilitation
Pediatric Balance Scale		18/56	40/56

**Figure 2:**
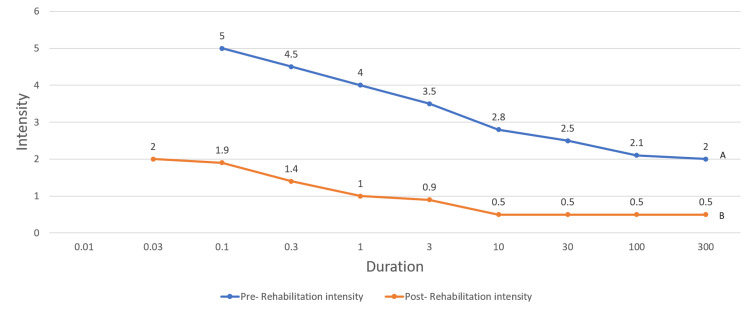
The strength-duration curve of tibialis anterior muscle pre- and post-rehabilitation

The strength-duration curve was plotted, which shows the status of innervations as depicted in Figure 2. She is under regular follow-up via telerehabilitation.

## Discussion

Iatrogenic sciatic nerve injury caused by a misdirected intramuscular injection in the gluteal region is a worldwide concern that impacts patients [3]. The sciatic nerve is most usually damaged by intramuscular injections administered in any quadrant, excluding the outer quadrant of the gluteal [11]. Nerve injuries sustained through intramuscular medication administration continue to impact rural populations more than urban populations. Injection nerve palsy has a typically positive prognosis [12]. A study performed by Senes et al. on a child with sciatic nerve injection palsy showed early microsurgical management and long-term results. They concluded that conservative treatment was successful [13].

Another study done by Fapojuwo et al. stated that sciatic nerve injury patients who are taking physiotherapy have significant improvement after the treatment, which increases functional capability and muscular strength [14]. The present case study is of traumatic injection palsy in a three-year-old female child whose parents reported that her child was unable to walk after receiving an intramuscular injection in the gluteal region for fever. As many studies show, physiotherapy plays an essential part in the recovery of injection nerve palsy following a foot drop. Also, the video-based home exercise programme increases the improvement, as shown in many articles [15]. Our study dealt with the role of the physiotherapy approach in a child with traumatic injection palsy. With electrical stimulation and the use of orthotic devices, coupled with therapeutic exercises, the foot drop can be well managed by therapists. We provided the patient with home rehabilitation exercises with video-based instruction and all exercise instructions were given to her parents. Physiotherapy helps in the improvement of strength, functional activities, gait pattern, and balance, prevents other deformities, and provides early and successful recovery.

## Conclusions

This case study shows that physiotherapy rehabilitation for injection palsy involving the sciatic nerve in a three-year-old female child with a foot drop led to improved lower-limb strength, which assisted the patient in ambulation and prevented other deformities. Hence, we can conclude that physiotherapy plays an essential part in the improvement of a patient's gait pattern, thus improving quality of life and the child’s body image perceptions. It also provides an early and successful recovery and manages the clinical manifestations of the condition.
